# Unusual Deep Water sponge assemblage in South China—Witness of the end-Ordovician mass extinction

**DOI:** 10.1038/srep16060

**Published:** 2015-11-05

**Authors:** Lixia Li, Hongzhen Feng, Dorte Janussen, Joachim Reitner

**Affiliations:** 1 State Key Laboratory of Palaeobiology and Stratigraphy, Nanjing Institute of Geology and Palaeontology, Chinese Academy of Sciences, Nanjing, 210008, China; 2 School of Earth Sciences and Engineering, Nanjing University, Nanjing, 210046, China; 3 Research Institute and Nature Museum Senckenberg, Frankfurt, 60325, Germany; 4 Department of Geobiology, Centre for Geosciences, Faculty of Geosciences and Geography, Georg-August-University of Göttingen, Göttingen, 37077, Germany

## Abstract

There are few sponges known from the end-Ordovician to early-Silurian strata all over the world, and no records of sponge fossils have been found yet in China during this interval. Here we report a unique sponge assemblage spanning the interval of the end-Ordovician mass extinction from the Kaochiapien Formation (Upper Ordovician-Lower Silurian) in South China. This assemblage contains a variety of well-preserved siliceous sponges, including both Burgess Shale-type and modern type taxa. It is clear that this assemblage developed in deep water, low energy ecosystem with less competitors and more vacant niches. Its explosion may be related to the euxinic and anoxic condition as well as the noticeable transgression during the end-Ordovician mass extinction. The excellent preservation of this assemblage is probably due to the rapid burial by mud turbidites. This unusual sponge assemblage provides a link between the Burgess Shale-type deep water sponges and the modern forms. It gives an excellent insight into the deep sea palaeoecology and the macroevolution of Phanerozoic sponges, and opens a new window to investigate the marine ecosystem before and after the end-Ordovician mass extinction. It also offers potential to search for exceptional fossil biota across the Ordovician-Silurian boundary interval in China.

The end-Ordovician mass extinction is the earliest and the second largest among the Big-five in Phanerozoic^1^. It occurred near the end of the Ordovician period (between 445.6 Ma and 443.7 Ma), and had a devastating influence on the marine ecosystem. It is estimated that about 85% of marine species became extinct during this crisis^2^. After nearly 40 years investigation in a global scale, there is a consensus that the end-Ordovician mass extinction was stepwise and episodic, and two phases have been recognized, with about one million years in between. Such biotic event was thought to be coincident with the Late Ordovician glaciation that took place mainly in Gondwana causing dramatic changes of global sea level and climate together with an oceanic turnover^2^,^3^,^4^,^5^. Many fossils have been found from the rocks chronostratigraphically corresponding to the interval from the first episode of the end-Ordovician mass extinction to the latest Ordovician, such as brachiopods, trilobites, corals, graptolites and so on^6^,^7^,^8^. Sponges are rare in this interval, and only lithistids and stromatoporoids have been sporadically documented^9^,^10^,^11^. Although many new discoveries show that the non-lithistid spicular sponges (especially the Burgess Shale-type faunas) are known from a broad range of times, and not restricted to the Cambrian, there are still large gaps in the fossil records of non-lithistid spicular sponges during the Late Ordovician^12^,^13^,^14^. So far, no sponges have been reported from the Late Ordovician in China. Fortunately, a new sponge assemblage bearing diverse and abundant well-preserved siliceous sponges was discovered from the Kaochiapien Formation (Upper Ordovician-Lower Silurian) of South China. It evolved slightly earlier than the second episode of the end-Ordovician mass extinction, flourished rapidly during the latest Hirnantian, and disappeared in South China at the beginning of the Silurian. This assemblage remarkably enhances our understanding of the marine ecosystem shortly before and after the end-Ordovician mass extinction event. With a brief discussion of the key taxa we also evaluated the relationship between our sponge assemblage and their modern counterpart in light of the morphological analysis, and explored the environmental background and taphonomic model of the sponge assemblage.

## Results

**Figure 1:**
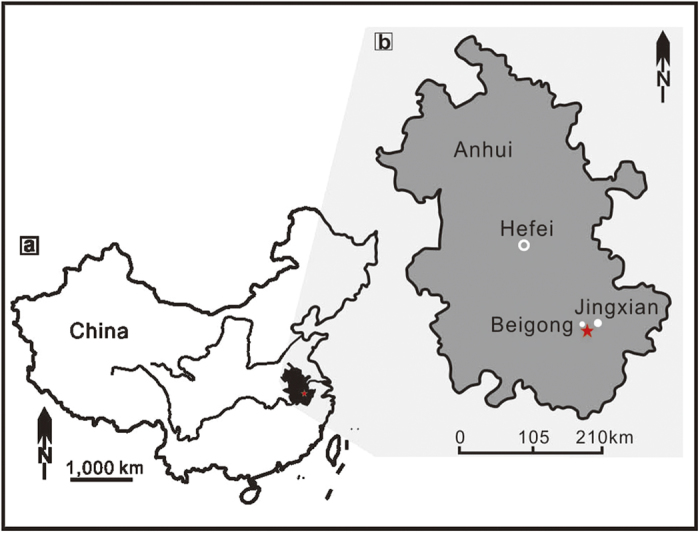
Locality map of Ordovician-Silurian boundary succession at Beigong, Anhui Province, South China. (**a**) Location of Anhui within China. (**b**) Location of studied section within Anhui indicated by a red star. Image created using CorelDRAW X5.

The sponge fossils have been collected in Kaochiapien Formation at Beigong of Jingxian County, southern Anhui Province, east China (Fig. 1). It is a continuous and fossiliferous Ordovician-Silurian boundary section with well-exposed outcrop. This locality is interpreted palaeogeographically to be part of the Lower Yangtze Platform, adjacent to the Xiang-Wan Basin^5^, characterized by graptolitic shale facies both near the top of Ordovician and the base of the Silurian. Abundant and diversified fossils were found at this section, including the well-preserved sponges and other groups such as graptolites, brachiopods, trilobites, cephalopods, echinoderms, ostracods, bivalves, and some indeterminate fossils. The sponge assemblage occurs mainly in the basal Kaochiapien Formation, which is characterized by black siliceous mudstone. The rocks show bluish-grey or greyish-white in color after weathering. The Kaochiapien Formation conformably overlies the Wufeng Formation that contains black siliceous and calcareous mudstone, yielding abundant shelly fossils such as brachiopods and trilobites.

According to the graptolites from the Wufeng and Kaochiapien formations, three graptolite biozones could be recognized in ascending order: *Metabolograptus extraordinarius* Biozone, *Metabolograptus persculptus* Biozone, and *Akidograptus ascensus* Biozone. The index fossils *M. persculptus* and *A. ascensus* are not found here, but other important species such as *Avitograptus avtitus*, *Normalograptus laciniosus*, *N. normalis*, *Neodiplograptus shanchongensis*, and *Ne. modestus* confirm the presence of the *M. persculptus* Biozone and *A. ascensus* Biozone at Beigong section.

**Figure 2:**
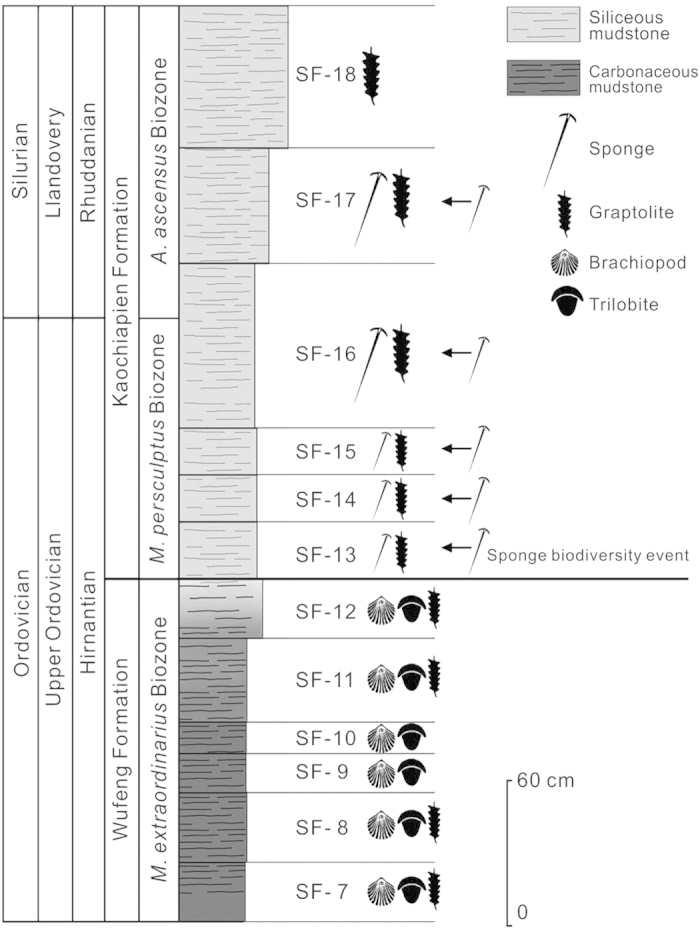
Outline of biostratigraphy (graptolite biozones) and lithostratigraphy of the Beigong section; the black arrows indicate horizons yielding sponge fossils. Image drawn by Lixia Li.

The sponges reported here belong to the *M. persculptus* Biozone and the lower part of the *A. ascensus* Biozone, indicating that they are of latest Ordovician (late Hirnantian) to earliest Silurian (early Rhuddanian) age, just extending across the Ordovician-Silurian boundary. But those shelly fossils (brachiopods and trilobites) are confined to the upper *M. extraordinarius* Biozone, suggesting a latest Ordovician (late Hirnantian) age (Fig. 2).

### Environmental interpretation

The presence of graptolites, brachiopods, trilobites, bivalves and echinoderms from the O-S transitional rocks suggests that the Beigong fauna is a typical Ordovician marine fauna. The core taxa of the *Hirnantia* fauna, *Aegiromena ultima*, *Paromalomena polonica*, and *Fardenia* sp., occur in our collection with low diversity and small sizes (between 5–10 mm), indicating an offshore, deep-water living environment with very poor nutrition supply^6^,^15^,^16^. The *Hirnantia-Dalmanitina* fauna at Beigong could be differentiated into three parts: the lower part is from the black calcareous mudstone (SF8-SF9), and characterized by rich *Dalmanitina* as well as one brachiopod (*Oxoplecia*? *incasfauta* Rong), representing a shallow water environment (about 20–30 m). There are no complete sponges found in this part, and only a few spicules are scattered in the rocks. The middle part is also from the black calcareous mudstone (SF10), but abundant in brachiopods together with a few trilobites. Brachiopods are dominated by *Aegiromena ultima*, typical of the *Paromalomena-Aegiromena* community with lower diversity and small shell sizes indicating a relatively deeper environment (about 60–150 m)^17^. No sponges were found in this part. The upper part is from the calcareous-siliceous mudstone (SF11-SF12), and is characterized by large trilobites (mainly *Platycoryphe*). *Dalmanitina* is also found in this part, but much less than in the lower part. The appearance of large trilobites shows that the oxygen level on the sea floor becomes higher during this interval^18^. Sponges are very rare in this part, with only a few scattered spicules in the rocks. Above these shelly beds, only sponges and graptolites were found, indicating a large scale transgression occurred during the interval when sponges flourished.

Overall, the Ordovician-Silurian transitional sequence at Beigong is not only characterized by shallow-water platform deposits (before the second pulse of the mass extinction) but also showing a hint of deep-water facies (after the second pulse of the mass extinction), which might be consistent with the unique palaeogeographic position of the section, stretching across the marginal Lower Yangtze Platform to the Xiang-Wan Basin. It is why the fossil assemblage at Beigong shows transitional affinity between platform and basin. It might be reasonable to infer that, during the latest Ordovician and the earliest Silurian, Beigong developed an open marine ecosystem on the platform margin, with relatively deep-water, low-energy, and oxygen-deficient conditions.

### Sponges

**Figure 3:**
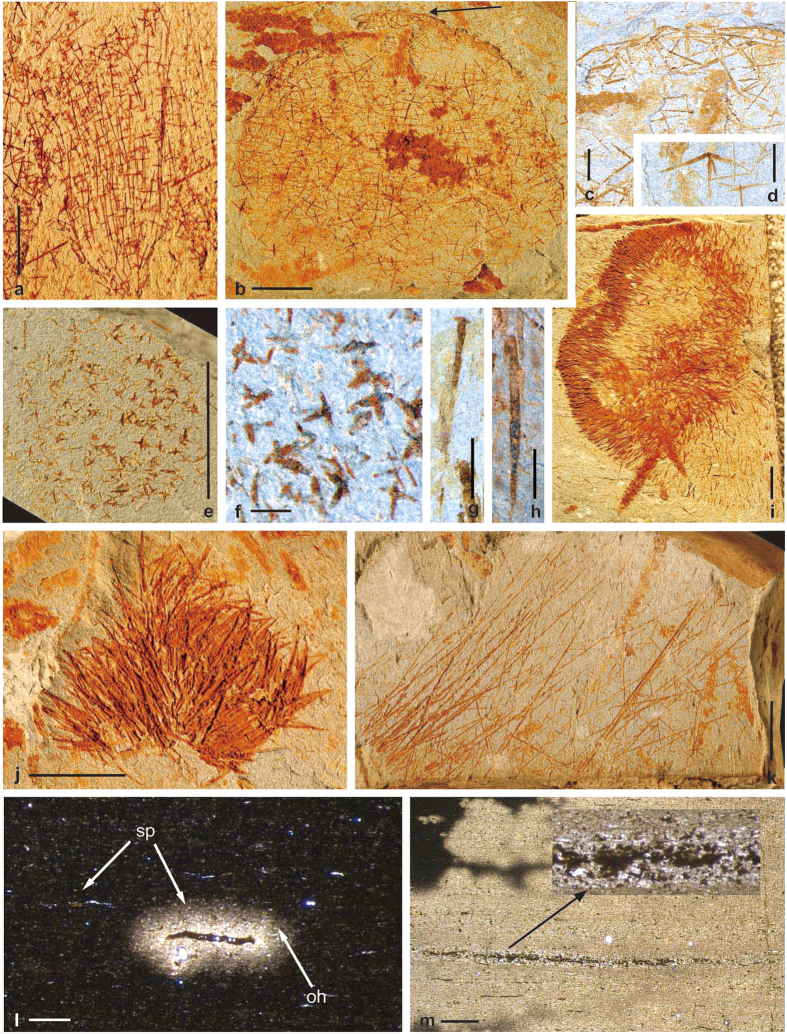
Representative sponges from the Beigong section. (**a**) SF14-26, Protospongiid showing typical budding behavior known from lyssakine Hexactinellida; (**b–d**) SF14-1, b, Hexasterophorid rossellimorph Hexactinellida with articulated stauracts and hexacts as well as characteristic pentactine dermal layer; (**c**) magnified view of (**b**) (black arrow), showing the characteristic pentactine dermal layer; (**d**) an isolate pentact spicule; (**e,f**), SF13-131, (**e**), *Thoracospongia* sp.; f is close up of e, showing inflated stauracts and swollen hexacts in varying sizes; (**g,i)**, SF13-124, (**g**), an isolate tetractinal spicule (triaene); (**i**), Cylindrical Tetractinellid demosponge preserved as oval compression with two layers of the body; (**h,j**), SF14-35, (**h**) an isolate monaxonal spicule (style); (**j**) Axinellid-type demosponge with plumose arrangement consisted of short styles; (**k**) SF14-8, Halichondrid-type demosponge with extremely long styles as *Dragmaxia*-type; (**l**) Autochthonous spiculite in the black shale (sp-spicules; oh-oxidation halo). (**m**) Autochthonous spiculite preserved in goethite after pyrite, the black arrow indicates spicule of the complete sponge. Scale bars: (**a,b,e,i–k**) 5 mm; (**c**), (**g,h**) 1 mm; d, f: 500 μm; (**l,m**) 200 μm. Photographs: Lixia Li (**a–k**), Joachim Reitner (**l,m**).

Diversified and well-preserved sponges occur in the black siliceous mudstone of the basal Kaochiapien Formation at Beigong. However, the spicules are preserved in late diagenetic iron hydroxides and end-product of pyrite oxidation. Therefore the spicules have lost some of their original form and structure. Pyrite precipitation as a product of microbial sulfate reduction in former spicule-moulds is characteristic of anaerobic sedimentary conditions. The sponges at Beigong systematically include two classes: Demospongiae (about 43% of the total) and Hexactinellida (about 57%). At least 15 types of articulated hexactinellids have been found, dominated by a few species in one family, with a more diverse assemblage of rare elements. The most abundant group is the vasiform or spheroidal hexactinellids, with thin body wall composed of regularly arranged stauractines (rare pentactines) parallel to the principal axis of the sponge body, usually forming quadrules in six orders, which is identified here as a new taxon of protospongids (Fig. 3a). The second group of hexactinellids is related to rossellimorph hexactinellids and is classified also as a new taxon, showing spherical growth form. Skeletal net consists of different types of hexactins, mainly stauracts paratangentalia in quadruled arrangement. Classical hexactins are also present. The outer margin is reinforced by pentactines with their four longer paratangential rays along the periphery, and the shorter one pointing toward to the center of the sponge body (Fig. 3b–d). Another group of hexactinellids with globular form is assigned to *Thoracospongia* Mehl, 1996^19^. Its spiculation consists mainly of irregularly oriented inflated stauractines and hexactines. The upper part of sponge body is composed of delicate spicules, while the basal part is reinforced by stout spicules, with a few delicate ones scattered sporadically (Fig. 3e,f).

Demosponges are also crucial component of this assemblage, with about 19 morphotypes having been found. According to spicule geometries and skeletal structures, two subclasses, Tetractinomorpha and Ceractinomorpha, have been recognized. The most distinctive group of Tetractinomorpha (Spirophorida) is identified as a new taxon closely related with modern tetillids, showing cylindrical form with walls of anatriaenes, triaenes, and long styles arranged in plumose pattern. Choanosomal skeleton is composed of randomly arranged anatriaenes and triaenes. Most of the individuals are preserved as oval compressions (Fig. 3g,i). A group of axinellid-type demosponges with hemispherical shape is composed of plumose arranged short styles, which radiate from the center of the sponge body, with all the pointed end of spicules pointing outward (Fig. 3h,j). Bundles of extremely long styles occur too, which are 80–120 μm in diameter, more or less grouped in fascicles. It was identified here as halichondrid-type Ceractinomorpha (Fig. 3k).

Although most of the specimens are articulated, a few dispersed siliceous spicules are also present, including monacts, diacts, stauracts, pentacts and hexacts (Fig. 3l,m). Only a small proportion of specimens are shown here. Detailed taxonomic description and phylogenetic interpretation are in progress and not the topic of the presented paper.

### Palaeoecological implication

The observed sponge assemblage is dominated by lyssakine “soft” hexactinellids (ca. 60%). Four major key taxonomic groups and related species were observed. Most of the specimens are articulated and show their pristine spicular inventory and anatomy. Therefore it was possible to determine the taxa in detail. The observed assemblage is a mixture of “old” Cambrian taxa (protospongids, thoracospongids) and modern-type rossellimorph hexactinellids. All of these hexactinellids are adapted to soft bottoms and known normally from low-energy deeper water settings. Rossellid hexactinellids are today common in deep water conditions often in cold polar environments^20^,^21^.

Also the spiculated demosponge assemblage exhibits a very modern character^22^. Surprisingly spirophorid demosponge taxa closely related to tetillids dominate the demosponge assemblage. It is the first occurrence of this demosponge taxon in the fossil record. Sponges with axinellid and halichondrid spicule arrangements are already known from the lower Cambrian (e.g. Chengjiang Biota)^23^. However, the forms discussed here are different and show morphological coincidences with modern type halichondrid/axinellid demosponges. Also these sponges are adapted to soft bottoms and low-energy environments. The entire sponge assemblage exhibits many coincidences with modern deep sea sponge communities and gives an excellent insight into the Late Ordovician deep sea palaeoecology.

## Discussion

As a group of sessile filter-feeding metazoans, sponges require relatively strict physical and chemical environment for their habitats. The mass disappearance of sponges during the first episode of the end-Ordovician mass extinction was thought to be related to the harsh climate and active upwelling with toxic components like enrichment of H_2_S^2^,^9^. Till early Silurian, sponges gradually recovered from the crisis in both richness and diversity^9^. Due to the sparse fossil record, it seems that there is an “evolutionary gap” of sponges from the first episode of the mass extinction to the beginning of Silurian. Fortunately, the sponges at Beigong are chronostratigraphically in this “gap”. They first appeared at the beginning of the *M. persculptus* Biozone, subsequent to the first extinction episode, then evolved quickly and flourished during and after the second pulse of the mass extinction. At the beginning of Silurian, the sponges disappeared rapidly. It should be noted that the sponges studied in this paper are only found at Beigong, southern Anhui, and no similar materials have been reported in any other places in South China and elsewhere in the world.

**Figure 4:**
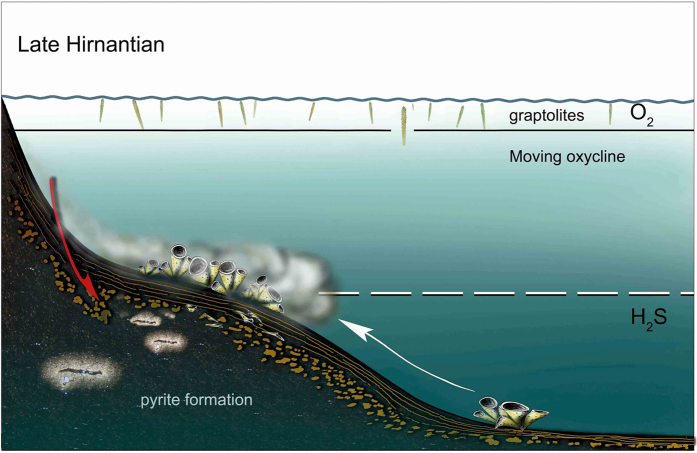
A conceptual model of the migration and taphonomic process of sponges during the late Hirnantian. The white arrow shows deep water sponge assemblage moves to relatively shallow water to escape from the anoxic and sulphidic water. The red arrow indicates the sponges are buried rapidly by mud turbidites (ocean chemistry model is after^27^). Image drawn by Lixia Li with the support of Joachim Reitner.

Why did sponges settle and flourish at Beigong during the end-Ordovician mass extinction? As mentioned above, Beigong had an environmental background of deeper water and low energy after the second pulse of the mass extinction, which was favorable to the siliceous sponges^24^. Besides, the diversity of marine ecosystem and the community types during the “survival-recovery interval” (i.e. the time between two pulses of the mass extinction) decreased sharply with much fewer predators and large numbers of nearly vacant niches^25^. In the Beigong area, only a few brachiopods and trilobites survived the first pulse of the mass extinction, leaving a unique opportunity for sponges to evolve and flourish during and after the second pulse of the mass extinction. Furthermore, a noticeable transgression is recognized to occur after the second pulse^26^ when sponges were flourishing in the Beigong area. So, it might be reasonable to propose that the explosion of sponges at Beigong should be related to this transgressive event. More recently, in light of analysis of geochemical redox indicators (such as iron speciation, molybdenum concentrations, pyrite framboid size distribution and sulfur isotopes), Hammarlund *et al.*^27^ suggested that the end-Ordovician mass extinction is linked to widespread euxinia together with near-global anoxia. In their model, during transgression in the late Hirnantian, the anoxic and weakly sulphidic water reached the continental shelves, affecting shallow benthos and causing extinction in shallower habitats^28^. Interestingly, this model is tested by the evolution of fauna and sedimentology from the Beigong section. After the second episode, the sea level rise triggered the migration of the deep anoxic water to the platform. This probably made the deep water sponge communities move to the relatively shallow water in order to “escape” from the new anoxic conditions (Fig. 4). It is suggested that the emergence of sponges in the marginal platform may be related to the expansion of the euxinic and anoxic condition. With transgression going on, sponge fauna disappeared, which may be triggered by the widespread anoxic and sulfidic water column conditions during the early Silurian time^27^,^29^.

The preservation of nearly complete sponge specimens is unusual and related to two taphonomic processes, i.e. low to zero oxygen in the deep water column and sediment in association with occasional fine-grained mud turbidites (described in detail by Einsele & Kelts^30^) which cover the sponges rapidly (Fig. 4). Mud turbidites exhibit sometimes little silt fraction at the base and thin laminated mud/clay-rich above. These types of thin (1–2 cm) turbidites are observed in close relationship with the sponge-layers. This taphonomic model is also recorded the Cambrian Lagerstätte Chengjiang Biota, which yields high diversity and well-preserved soft-bodied fossils. As noted by Zhao *et al.*^31^, the exceptional preserved fossils of Chengjiang Biota were smothered by storm-generated mud clouds and buried rapidly with low bioturbation and low hydrodynamic disturbance. It shows couplets of thin layers in their mudstone samples, which were supposed to be subjected to different taphonomic processes, looking similar to our thin layer mud turbidite sediment. This kind of taphonomic model prevents the pre- or syn-burial decay and promotes the complete preservation both in Chengjiang and Beigong.

## Methods

All figured specimens are deposited in Paleontology and Stratigraphy Laboratory, Department of Earth Science, Nanjing University, and indicated by their accession numbers. Locality details for all the fossils are saved in Nanjing University, and can be provided by the authors upon request. Photographs were made with SONY A350 digital reflex camera, and Zeiss SteREO Discovery V8 stereomicroscope (transmitted- and reflected light) linked to an AxioCam MRc 5-megapixel camera. Figures 2 and 4 were conducted by using Drawing tablet (WACOM, Intuos, PTK-840). Digital photographs were processed in Adobe Photoshop CS3 and composite images were stitched together using CorelDraw X5.

## Additional Information

**How to cite this article**: Li, L. *et al.* Unusual Deep Water sponge assemblage in South China—Witness of the end-Ordovician mass extinction. *Sci. Rep.* **5**, 16060; doi: 10.1038/srep16060 (2015).
